# Technical feasibility of leadless left bundle branch area pacing for cardiac resynchronization: a case series

**DOI:** 10.1093/ehjcr/ytab379

**Published:** 2021-09-24

**Authors:** Mark K Elliott, Peggy Jacon, Baldeep Singh Sidhu, Lucy Jarrett Smith, Vishal S Mehta, Justin Gould, Angela W C Lee, Steven Niederer, Pascal Defaye, Christopher A Rinaldi

**Affiliations:** 1 School of Biomedical Engineering and Imaging Sciences, King’s College London, St Thomas' Hospital, Westminster Bridge Road, London, SE1 7EH, UK; 2 Department of Cardiology, St Thomas' Hospital, Westminster Bridge Road, London, SE1 7EH, UK; 3 Arrhythmias Unit, Department of Cardiology, Grenoble Alpes University Hospital, 38700 La Tronche, Grenoble, France

**Keywords:** Cardiac resynchronization therapy, Endocardial pacing, Left bundle branch area pacing, Leadless pacing, Heart failure, Case series

## Abstract

**Background:**

Left bundle branch area pacing (LBBAP) is a novel form of conduction system pacing which can reverse left bundle branch block and deliver cardiac resynchronization therapy (CRT). The WiSE-CRT system delivers leadless endocardial pacing with symptomatic and left ventricular (LV) remodelling improvements following intervention. We report the technical feasibility of delivering leadless LBBAP using the WiSE-CRT system.

**Case summary:**

In Case 1, a 57-year-old male with ischaemic cardiomyopathy and complete heart block underwent implantation of the WiSE-CRT system, using a retrograde transaortic approach, after failed conventional CRT. Temporary left bundle stimulation from the LV septum achieved superior electrical resynchronization and equivalent haemodynamic response compared to endocardial pacing at the lateral LV wall. In Case 2, an 82-year-old gentleman with tachyarrhythmia-induced cardiomyopathy underwent WiSE-CRT implantation via a trans-septal inter-atrial approach, with the endocardial electrode successfully deployed in the LV septum.

**Discussion:**

Here we report the first case of deployment of the WiSE-CRT endocardial electrode in the LV septum and demonstrate the technical feasibility of leadless LBBAP. Entirely leadless CRT is an attractive option for patients with venous access issues or recurrent lead complications and has previously been successful using the WiSE-CRT system and a leadless pacemaker in the right ventricle. Further studies are required to assess long-term efficacy and safety of leadless LBBAP.

Learning points

- Left bundle branch area pacing (LBBAP) from the left ventricular (LV) aspect of the septum achieves excellent electrical resynchronization and haemodynamic response.
- Deployment of the WiSE-CRT endocardial electrode in the LV septum is feasible.
- This demonstrates the technical feasibility of leadless LBBAP using the WiSE-CRT system.

## Introduction

**Figure 1:**
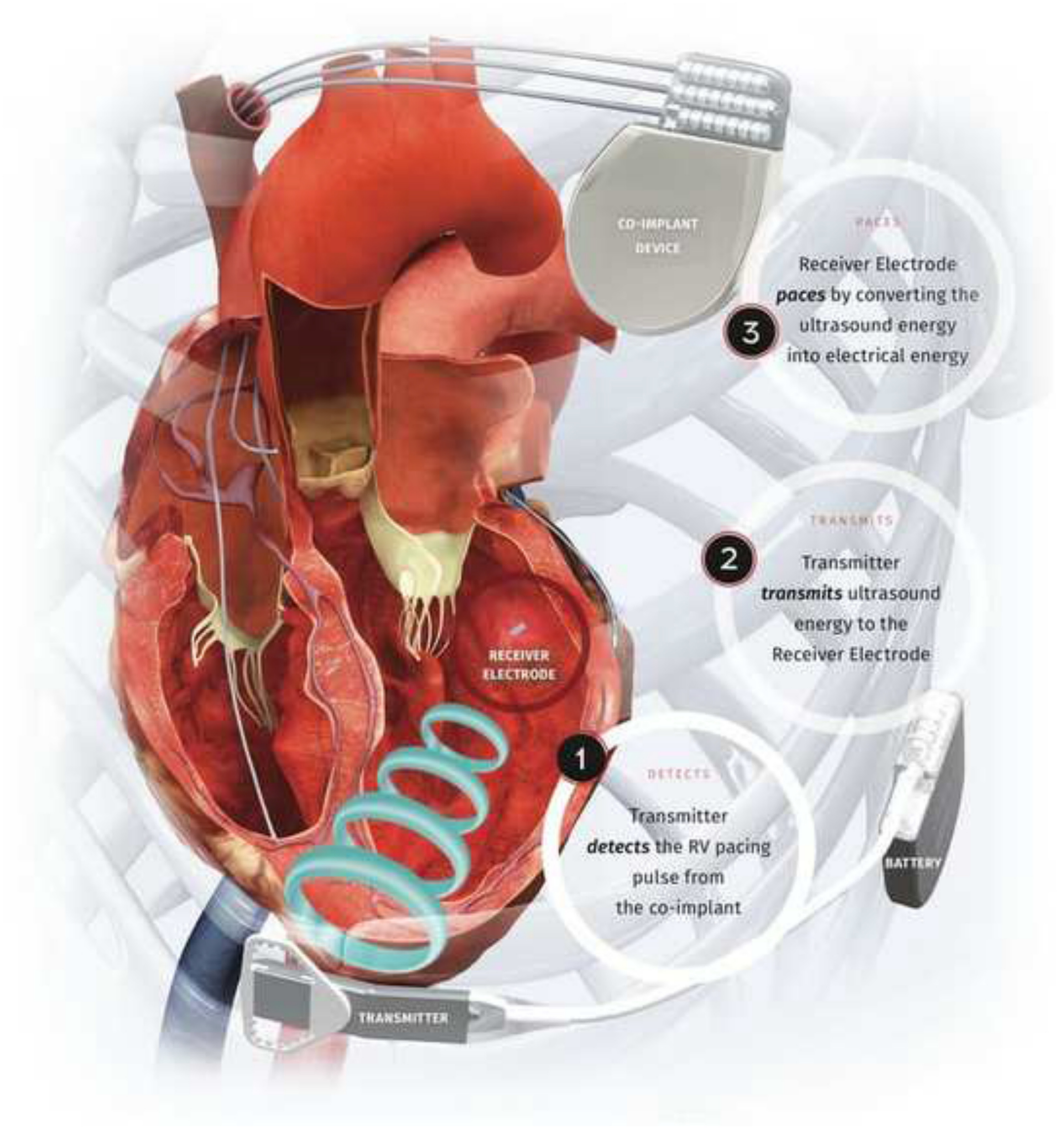
Components of the WiSE-CRT system (reproduced with permission from EBR Systems). RV, right ventricular.

Left bundle branch area pacing (LBBAP) has been proposed as an alternative to His bundle pacing (HBP), and has been shown to reverse left bundle branch block (LBBB) and deliver cardiac resynchronization therapy (CRT) in observational studies.^1–3^ The WiSE-CRT system (EBR Systems, Sunnyvale, CA, USA) can deliver leadless left ventricular (LV) pacing and has been shown to improve symptoms and LV remodelling in CRT non-responders.^4^^,^^5^ The components of the WiSE-CRT system are demonstrated in *Figure  1*. After pre-procedural acoustic screening to identify an appropriate intercostal space, patients undergo implantation of the transmitter over the intercostal muscle and implantation of the generator placed in the adjacent mid-axillary line. The endocardial electrode is implanted either on the same sitting or as a separate procedure in a two-stage technique. Implantation can be performed via a retrograde aortic approach using femoral arterial access or via an inter-atrial trans-septal approach using femoral venous access. The system requires the presence of a co-implant capable of delivering continuous right ventricular (RV) pacing. After identification of the RV pacing signal, the transmitter delivers a focused beam of ultrasound energy to the endocardial electrode, which converts this into electrical energy to capture to the LV myocardium and achieve biventricular pacing. We describe two cases of patients undergoing leadless LV endocardial pacing following failed conventional CRT. In the first case, temporary stimulation of the LV septum achieved optimal electrical and haemodynamic indices. In the second case, the endocardial electrode was successfully deployed in the LV septum. These cases demonstrate the feasibility of conduction system pacing by leadless stimulation of the left bundle branch.

## Timeline

**Figure:**
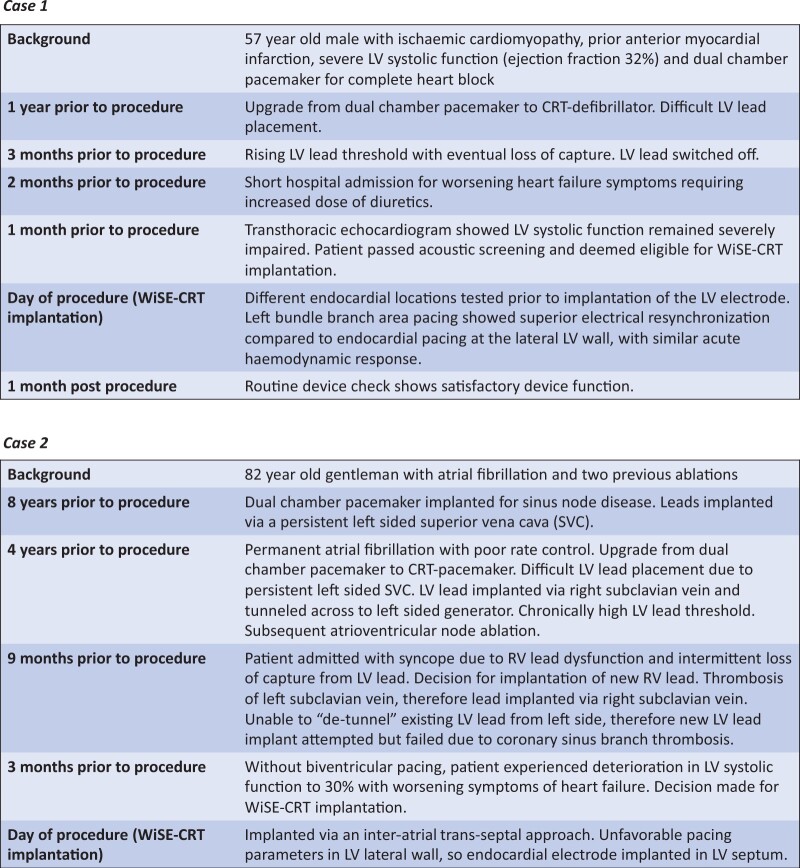


## Case presentations

### Case 1

A 57-year-old male with ischaemic cardiomyopathy, prior anterior myocardial infarction, and a dual-chamber pacemaker for complete heart block underwent upgrade to a CRT-defibrillator. He had New York Heart Association (NYHA) Class III symptoms and severe LV systolic impairment (ejection fraction 32%) despite optimal medical therapy. Physical examination findings were in keeping with chronic heart failure. The upgrade procedure was difficult due to small calibre coronary sinus (CS) tributaries. The LV lead was placed in a lateral branch of the CS, however, during routine follow-up the lead threshold increased with eventual loss of capture. Further transvenous attempts were unlikely to be successful given the difficulty of the original procedure and so the patient underwent leadless endocardial pacing using the WiSE-CRT system.

**Figure 2:**
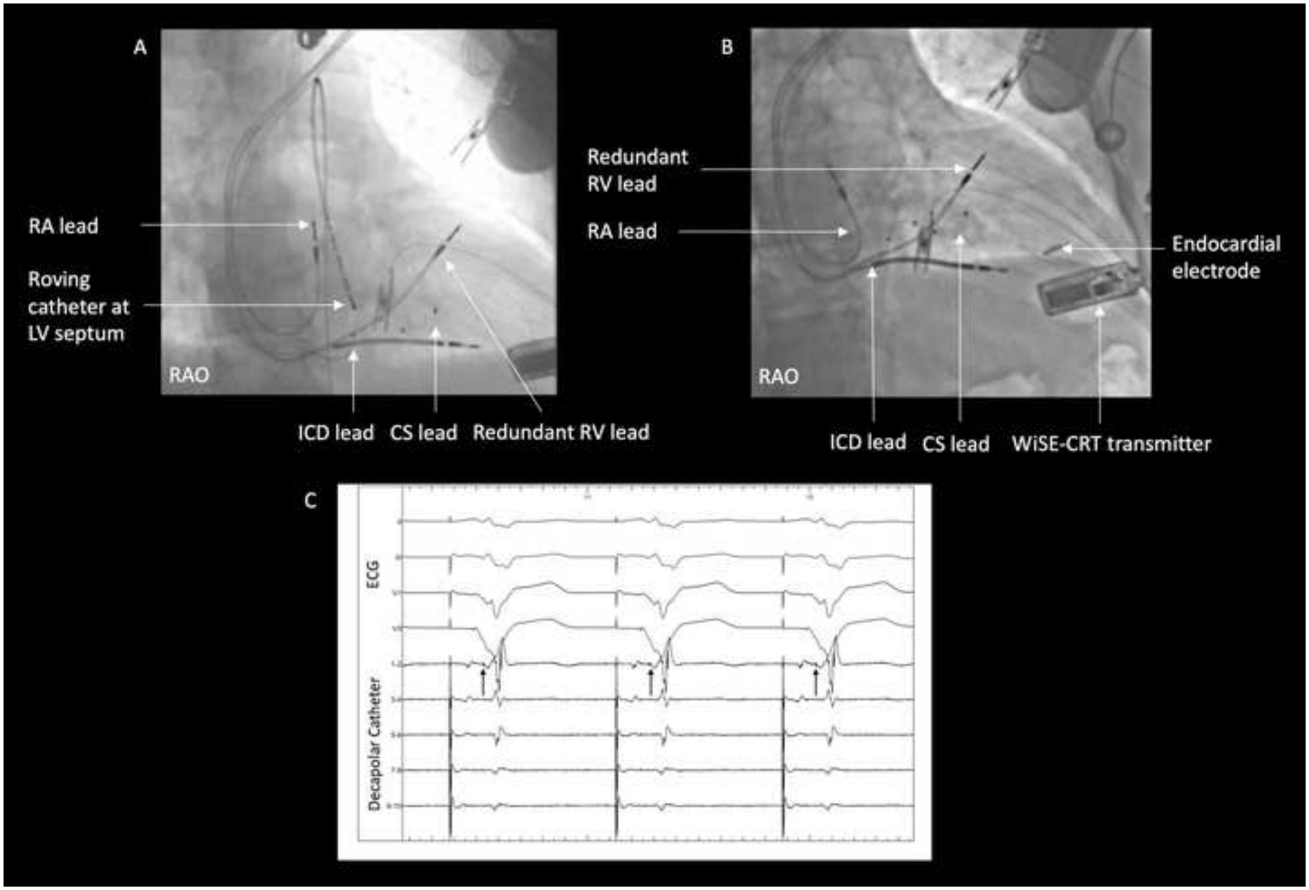
(Case 1) (*A*) Catheter positions during temporary left bundle branch area pacing. (*B*) Final endocardial electrode placement in mid-lateral wall of the left ventricle. (*C*) Surface electrocardiogram and intracardiac electrograms from the roving decapolar catheter during baseline dual-chamber right ventricular pacing. The decapolar catheter is positioned on the LV aspect of the septum and dipoles are ordered from distal (1-2) to proximal (9-10).^7^^,^^8^ The retrograde left bundle potential is marked with arrows on poles 1–2. Sweep speed 100 mm/s. CS, coronary sinus; ECG, electrocardiogram; ICD, implantable cardioverter-defibrillator; LV, left ventricular; RAO, right anterior oblique; RA, right atrial; RV, right ventricular.

**Figure 3:**
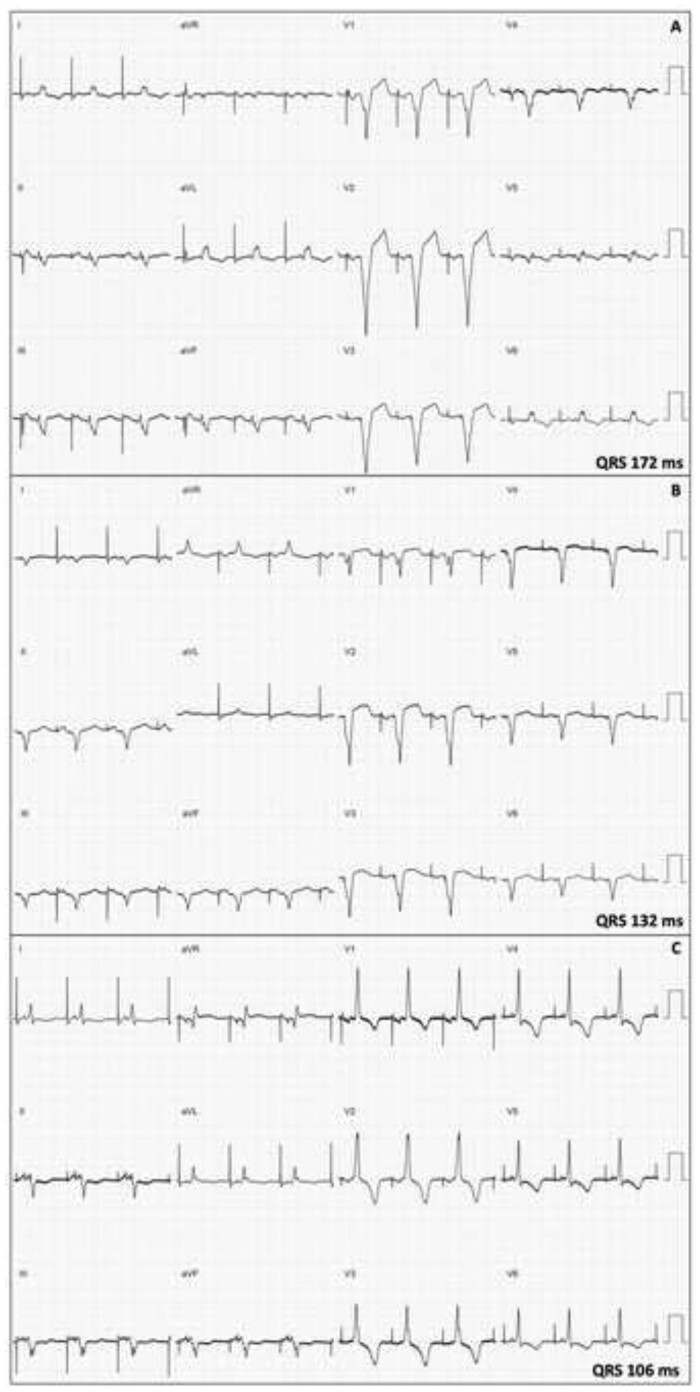
(Case 1) Surface electrocardiograms during (*A*) right ventricular pacing; (*B*) biventricular pacing with electrode at mid-lateral wall of left ventricle; and (*C*) biventricular pacing with electrode at the left bundle branch area.

The WiSE-CRT system was implanted using a previously described technique with a retrograde transaortic approach.^4^ Prior to implantation of the LV electrode, different myocardial locations were tested using a roving decapolar catheter (6-F Livewire 115 cm, St Jude Medical, Inc., St Paul, MN, USA) to assess for the optimal acute haemodynamic response (AHR) and paced QRS duration. Temporary biventricular pacing was achieved by pacing the LV endocardium at the same atrioventricular delay as patient’s existing pacemaker. Haemodynamic assessment (LV dP/dt_max_) was performed with a pressure wire in the LV cavity using a previously described protocol.^6^ Left ventricular dP/dt_max_ measurements were recorded using CoroFlow (Coroventis, Uppsala, Sweden) and AHR was expressed as percentage improvement from baseline dual-chamber RV pacing to biventricular pacing at different LV endocardial sites. Left bundle branch area pacing was achieved by pacing the LV aspect of the interventricular septum (*Figure  2A*) at the site of a left bundle potential (*Figure  2C*). The greatest AHR was seen when pacing the left bundle (34% increase from baseline) and was equivalent to endocardial stimulation at the mid-lateral wall (34%) as shown in *Table 1*. However, LBBAP showed greater electrical resynchronization (QRS duration 106 ms) compared to pacing at the mid-lateral LV wall (QRS 132 ms) and baseline RV pacing (172 ms) as shown in *Figure  3*. The endocardial electrode was deployed in the mid-lateral wall of the LV as the current WiSE-CRT system is not designed to target the septum when a retrograde aortic approach is used (*Figure  2B*).

**Table 1:** (Case 1) Acute haemodynamic response with biventricular pacing for different left ventricular pacing locations, expressed as a percentage increase in dP/dTmax compared to baseline right ventricular pacing

Pacing location in left ventricle	Acute haemodynamic response (%)
Basal lateral	27
Mid-lateral	34
Apical septum	21
Left bundle branch	34

### Case 2

**Figure 4:**
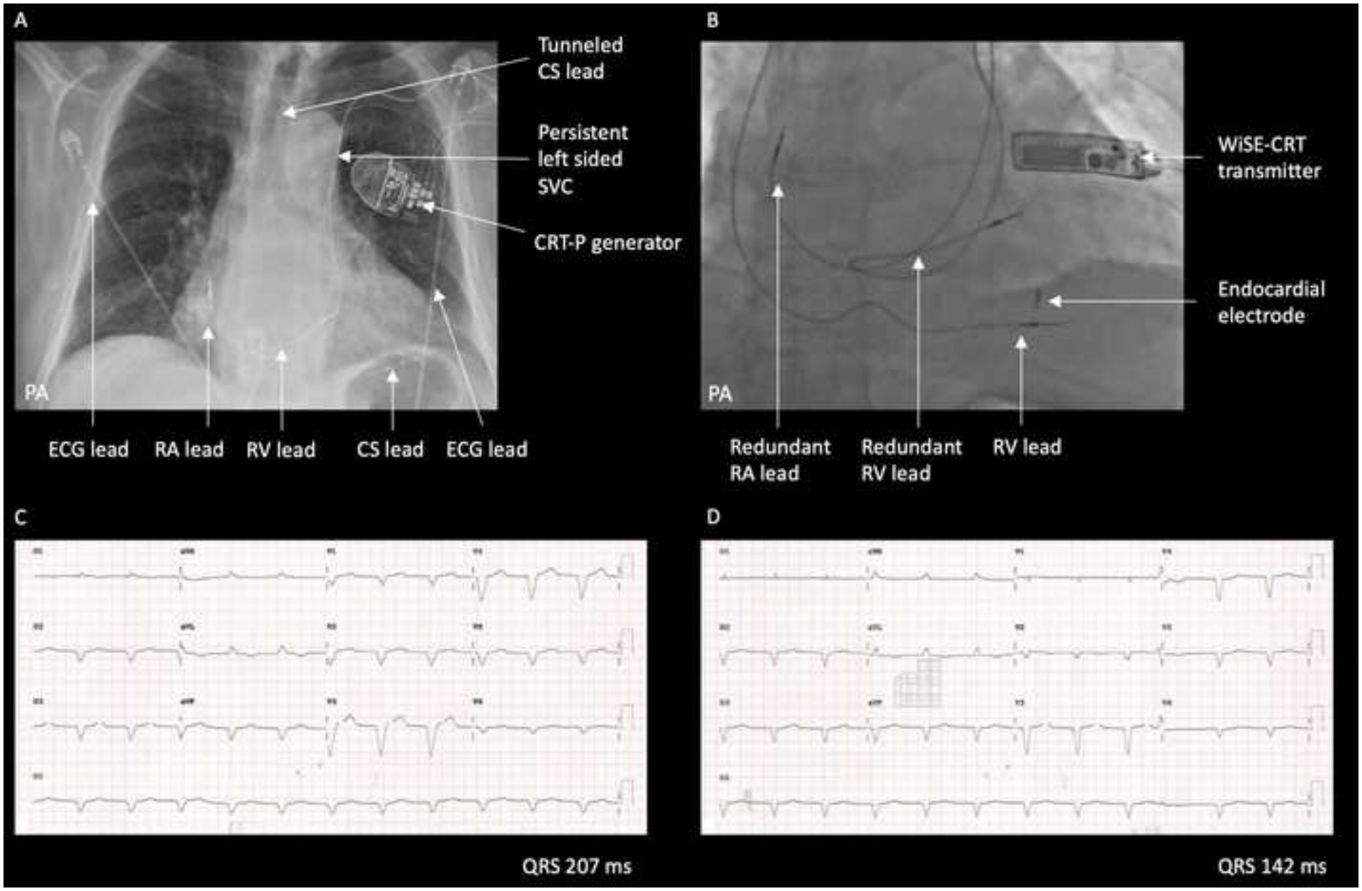
(Case 2) Electrode and lead positions on (*A*) chest X-ray prior to WiSE-CRT implant and (*B*) intra-procedure fluoroscopy after WiSE-CRT implant. Surface electrocardiograms during right ventricular pacing (*C*) and biventricular pacing with the endocardial electrode in the left ventricular septum (*D*). CRT-P, cardiac resynchronization therapy pacemaker; CS, coronary sinus; ECG, electrocardiogram; PA, posteroanterior; RA, right atrial; RV, right ventricular; SVC, superior vena cava.

An 82-year-old gentleman with tachyarrhythmia-induced cardiomyopathy required upgrade to a CRT-pacemaker prior to undergoing an atrioventricular node ablation for poorly controlled atrial fibrillation. He had an existing dual-chamber pacemaker which was implanted 4 years previously for sinus node disease, with leads placed via a persistent left-sided superior vena cava (SVC). LV lead placement in the CS was difficult due to the left-sided SVC, and was ultimately implanted via right subclavian access and tunnelled across to the left-sided generator (*Figure  4A*). Four years later the patient was re-admitted with syncope associated with RV lead dysfunction and a high LV lead threshold with intermittent loss of capture. The left subclavian vein was occluded, and so a new RV lead was implanted via the right subclavian vein. It was not possible to transfer the previously tunnelled LV lead to the right side, and so the existing LV lead was extracted. Re-implantation of a new LV lead failed due to thrombosis of the posterolateral branch of the CS. Without biventricular pacing, the patient deteriorated with worsening LV systolic function (ejection fraction 30%) and heart failure symptoms (NYHA Class III) despite optimal medical therapy. Physical examination findings were in keeping with chronic heart failure. The patient subsequently underwent implantation of the WiSE-CRT system, which was performed via a trans-septal inter-atrial approach using the FlexCath Advance Steerable Sheath (Medtronic Inc., Minneapolis, MN, USA). Sensing and pacing threshold measurements in the LV lateral wall were not acceptable and so the endocardial electrode was implanted in the LV septum (*Figure  4B* and *Video 1*) with significantly improved electrical resynchronization on electrocardiogram (*Figure  4C and D*). A stable LV capture threshold (1.5 V at 0.5 ms) and high biventricular pacing percentage (98%) were reported on the pre-discharge device check. At 6-month follow-up, device function was satisfactory and the patient reported significant symptomatic improvement (NYHA Class II).

## Discussion

Both conventional (epicardial) and endocardial CRT deliver two non-physiological wavefronts which merge to resynchronize the myocardium. Conduction system pacing can recruit the intrinsic His–Purkinje system and reverse LBBB. His bundle pacing is feasible in delivering CRT in heart failure patients, with electrical resynchronization and AHR superior to conventional CRT.^9^ However, it may be limited by elevated pacing thresholds at follow-up and in a recent registry study, loss of His bundle capture was found in 17% during a 2-year follow-up period.^10^ Left bundle branch area pacing has been recently proposed as a method to reverse LBBB at lower thresholds^1–3^ and is usually achieved with delivery tools and techniques for HBP, with the electrode fixed deep in the interventricular septum via an RV approach. The ability to perform temporary LBBAP via a retrograde transaortic approach has been demonstrated, with favourable electrical resynchronization;^7^ however, permanent placement of a lead to the LV septum is not feasible due to the risk of embolic stroke. The WiSE-CRT system does not suffer from this drawback as the device becomes fully endothelialized and therefore does not pose a long-term risk of embolism.

In Case 1, temporary LBBAP was associated with excellent electrical resynchronization (QRS duration 106 ms) and haemodynamic indices (AHR 34%). However, the endocardial electrode was ultimately deployed in the LV lateral wall as the current WiSE-CRT delivery system is not designed to target the septum when a retrograde transaortic approach is used, and rotation of the delivery catheter to reach the septum is technically challenging. In Case 2, a trans-septal inter-atrial approach to the LV was used, which allowed successful deployment of the endocardial electrode in the septum. This is the first reported case of LBBAP delivered via the WiSE-CRT system and, to the best of our knowledge, this is the first reported case of permanent LBBAP from the LV aspect of the interventricular septum. Left ventricular septal mapping was not performed to identify the specific location of a left bundle branch potential in Case 2, and therefore selective stimulation of the left bundle branch cannot be guaranteed, however, the marked reduction in QRS duration (from 207 ms during RV pacing to 142 ms during biventricular pacing) suggests that at least non-selective stimulation of the left bundle branch was achieved. Comparison of the QRS morphology during LBBAP via the WiSE-CRT system with that of previously reported lead-based LBBAP^8^ is difficult as the former requires continuous RV pacing. The QRS morphology therefore represents a fusion of pacing from the RV and the left bundle branch area.

Entirely leadless CRT systems are an attractive option in patients with vascular access issues, such as haemodialysis patients, and in those with recurrent lead complications. While the majority of WiSE-CRT systems are implanted in patients with standard right-sided pacing systems, there have been reports of entirely leadless systems using the WiSE-CRT system in conjunction with a leadless pacemaker in the RV.^11^ This case series demonstrates the feasibility of leadless LBBAP using the WiSE-CRT system, particularly when a trans-septal approach to the LV is used. It should be noted that the endocardial electrode has not been specifically designed to ensure that the 3.6 mm tines will sufficiently penetrate the endocardial surface down to the Purkinje tissue within the septum. However, the left bundle branch sits closer to the LV aspect of the septum, and LBBAP from an RV approach requires deep penetration into the septum, with a reported range of 11–18 mm in an observational study of 100 patients.^12^ It is therefore likely that an LV approach requires more superficial penetration, but further evaluation is needed.

In conclusion, in the first case, electrical resynchronization appeared superior during temporary LBBAP, compared to endocardial pacing in the lateral LV wall, with similar haemodynamic responses. In the second case, the endocardial electrode was successfully deployed in the LV septum and is the first reported case of LBBAP via the WiSE-CRT system. Together, these cases demonstrate the feasibility of leadless LBBAP. Further studies are required to assess long-term safety and performance.

## Lead author biography 

**Figure:**
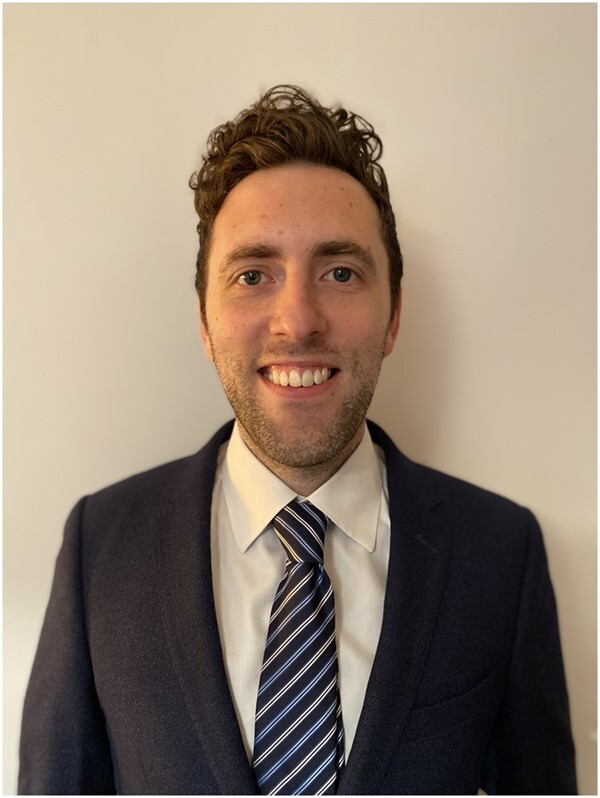


Mark Elliott is a cardiology trainee in the North East Thames deanery and a PhD research fellow working in the school of Biomedical Engineering and Imaging Sciences at King’s College London. His research interests include cardiac resynchronization therapy, endocardial left ventricular pacing, conduction system pacing and electrocardiographic imaging.

## Supplementary material

Supplementary material is available at *European Heart Journal - Case Reports* online.

## Supplementary Material

ytab379_Supplementary_Data
