# Supplementary material for: Technical feasibility of leadless left bundle branch area pacing for cardiac resynchronization: a case series
Source: Eur Heart J Case Rep. 2021 Sep 24;5(11):ytab379. doi: 10.1093/ehjcr/ytab379 (PMC8633604; doi:10.1093/ehjcr/ytab379)
Supplement: ytab379_Supplementary_Data [file ytab379_supplementary_data.zip › WiSE_CR_Slide_Set.pptx]

## Slide 1
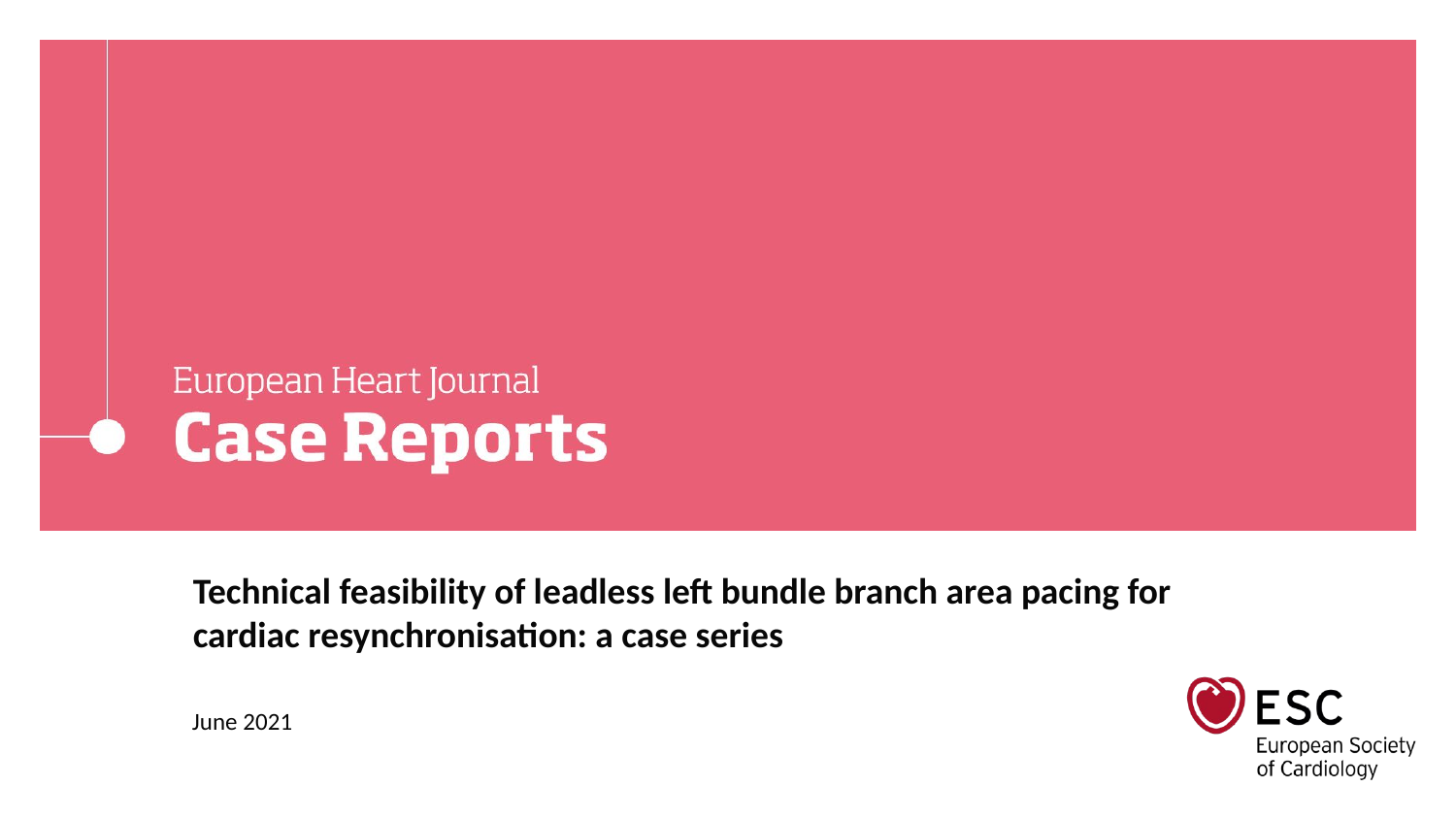

# Technical feasibility of leadless left bundle branch area pacing for cardiac resynchronisation: a case series
June 2021

## Slide 2
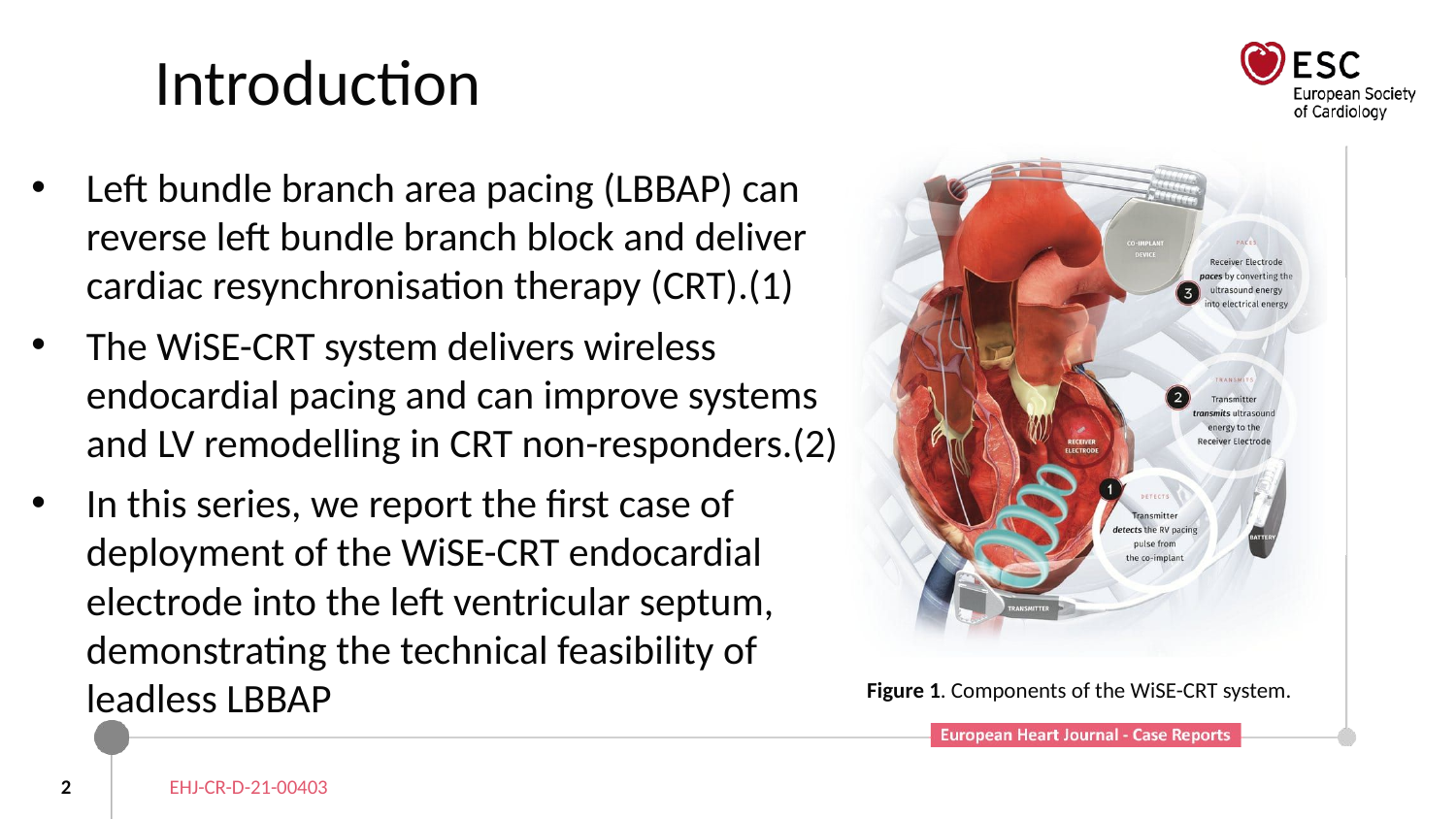

# Introduction
Left bundle branch area pacing (LBBAP) can reverse left bundle branch block and deliver cardiac resynchronisation therapy (CRT).(1)
The WiSE-CRT system delivers wireless endocardial pacing and can improve systems and LV remodelling in CRT non-responders.(2)
In this series, we report the first case of deployment of the WiSE-CRT endocardial electrode into the left ventricular septum, demonstrating the technical feasibility of leadless LBBAP
Figure 1. Components of the WiSE-CRT system.
2
EHJ-CR-D-21-00403

## Slide 3
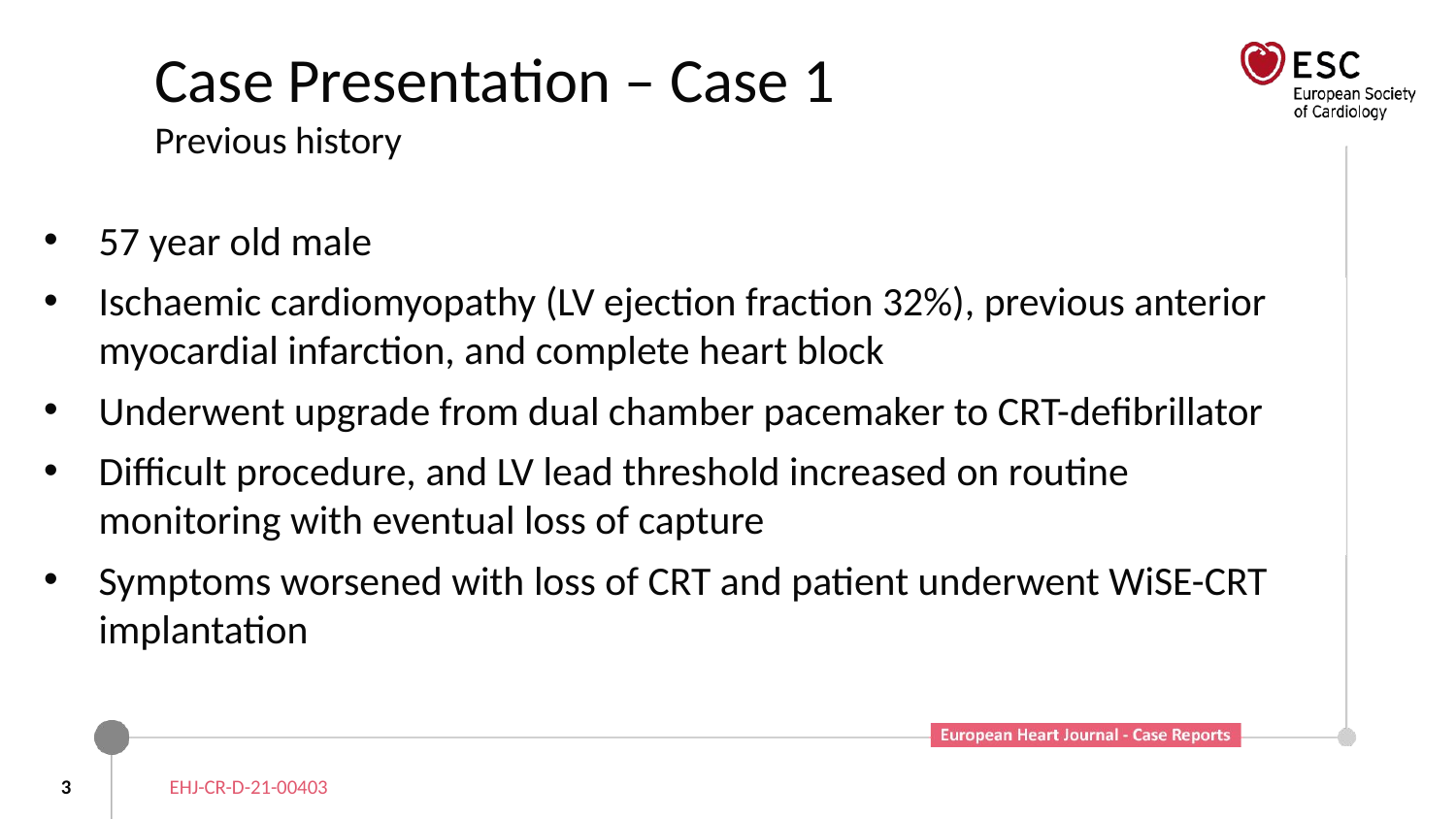

# Case Presentation – Case 1 Previous history
57 year old male
Ischaemic cardiomyopathy (LV ejection fraction 32%), previous anterior myocardial infarction, and complete heart block
Underwent upgrade from dual chamber pacemaker to CRT-defibrillator
Difficult procedure, and LV lead threshold increased on routine monitoring with eventual loss of capture
Symptoms worsened with loss of CRT and patient underwent WiSE-CRT implantation
3
EHJ-CR-D-21-00403

## Slide 4
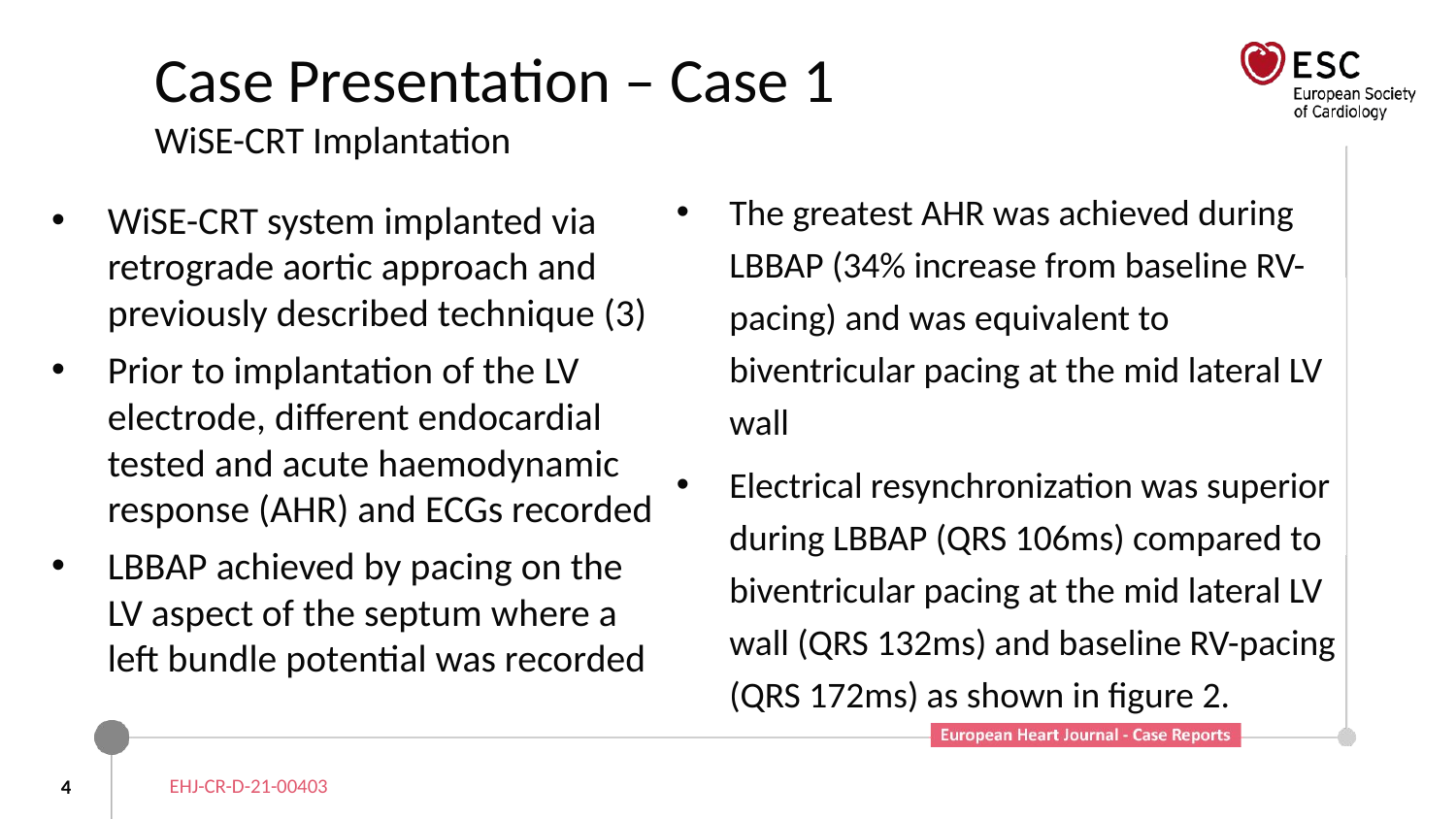

# Case Presentation – Case 1 WiSE-CRT Implantation
The greatest AHR was achieved during LBBAP (34% increase from baseline RV-pacing) and was equivalent to biventricular pacing at the mid lateral LV wall
Electrical resynchronization was superior during LBBAP (QRS 106ms) compared to biventricular pacing at the mid lateral LV wall (QRS 132ms) and baseline RV-pacing (QRS 172ms) as shown in figure 2.
WiSE-CRT system implanted via retrograde aortic approach and previously described technique (3)
Prior to implantation of the LV electrode, different endocardial tested and acute haemodynamic response (AHR) and ECGs recorded
LBBAP achieved by pacing on the LV aspect of the septum where a left bundle potential was recorded
EHJ-CR-D-21-00403
4

## Slide 5
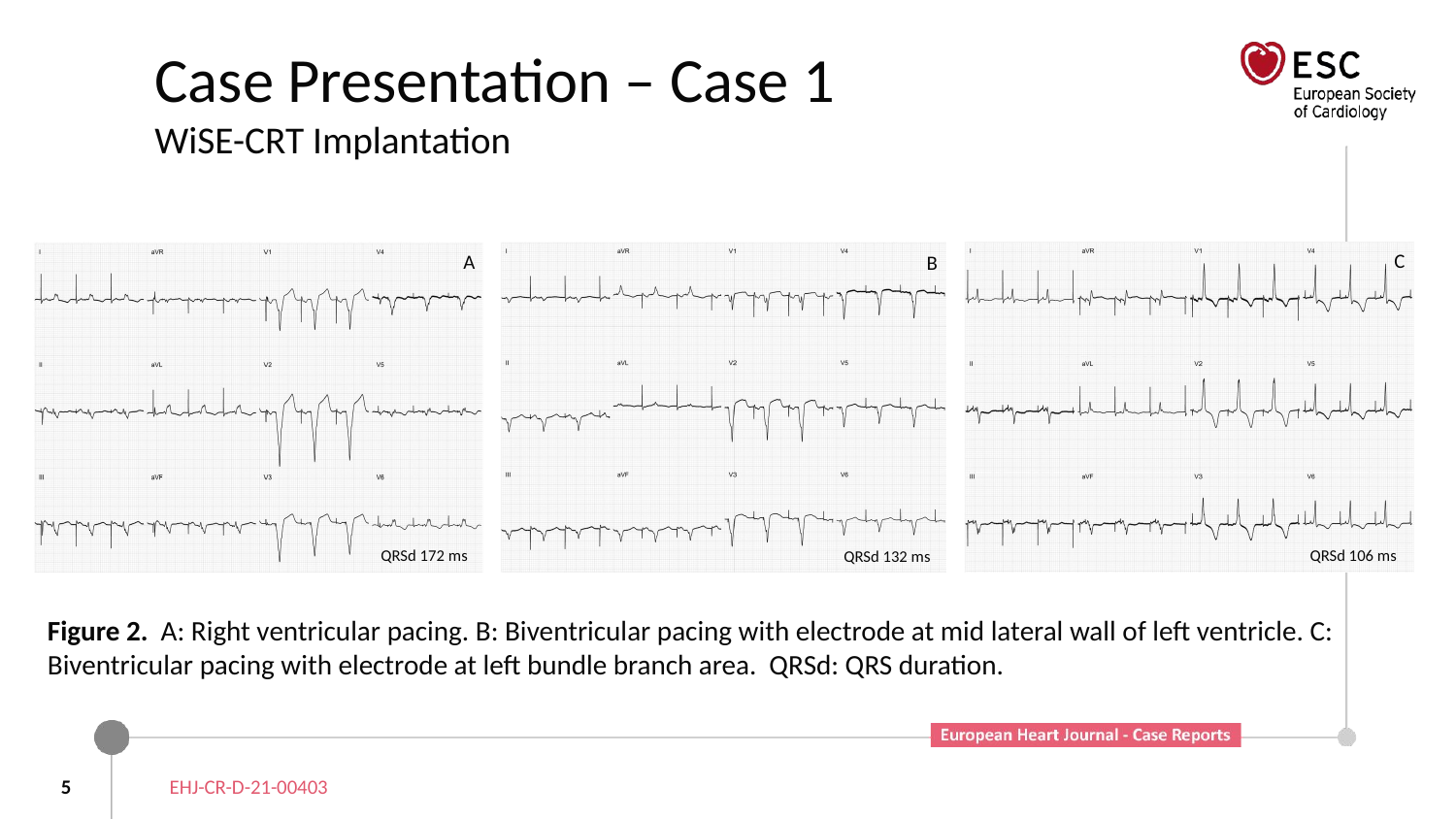

# Case Presentation – Case 1 WiSE-CRT Implantation
C
A
B
QRSd 172 ms
QRSd 106 ms
QRSd 132 ms
Figure 2. A: Right ventricular pacing. B: Biventricular pacing with electrode at mid lateral wall of left ventricle. C: Biventricular pacing with electrode at left bundle branch area. QRSd: QRS duration.
5
EHJ-CR-D-21-00403

## Slide 6
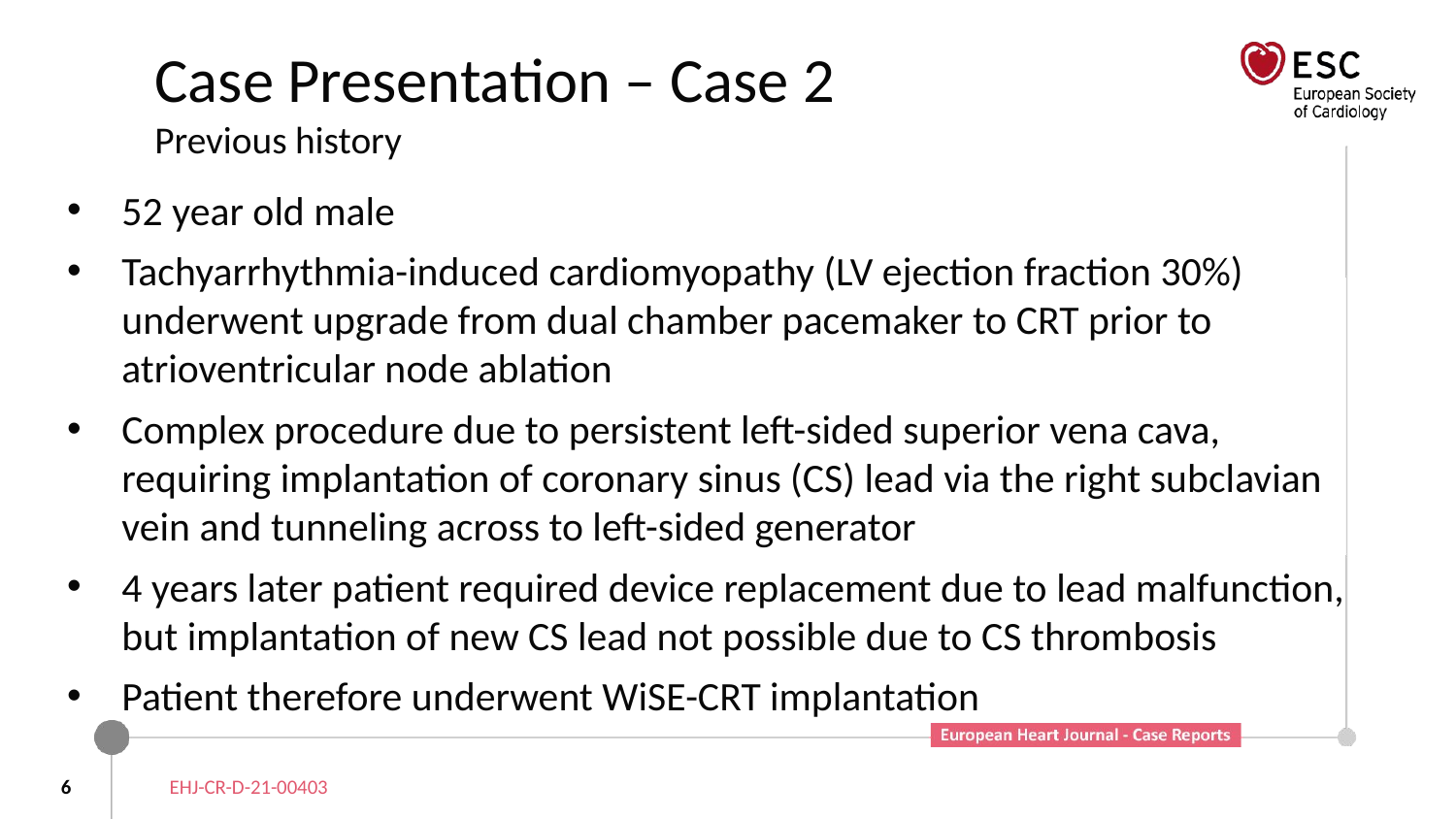

# Case Presentation – Case 2 Previous history
52 year old male
Tachyarrhythmia-induced cardiomyopathy (LV ejection fraction 30%) underwent upgrade from dual chamber pacemaker to CRT prior to atrioventricular node ablation
Complex procedure due to persistent left-sided superior vena cava, requiring implantation of coronary sinus (CS) lead via the right subclavian vein and tunneling across to left-sided generator
4 years later patient required device replacement due to lead malfunction, but implantation of new CS lead not possible due to CS thrombosis
Patient therefore underwent WiSE-CRT implantation
6
EHJ-CR-D-21-00403

## Slide 7
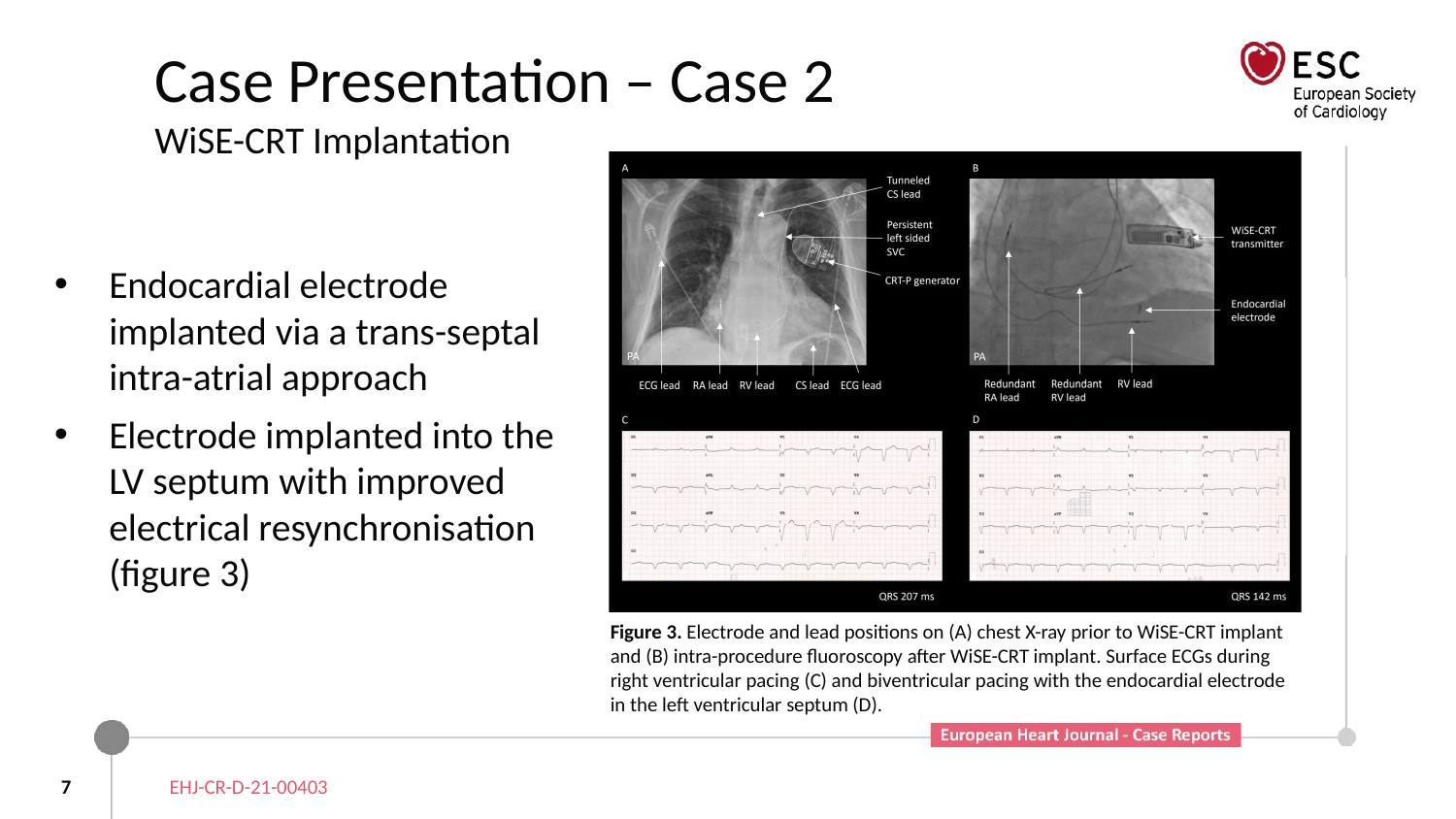

# Case Presentation – Case 2 WiSE-CRT Implantation
Endocardial electrode implanted via a trans-septal intra-atrial approach
Electrode implanted into the LV septum with improved electrical resynchronisation (figure 3)
Figure 3. Electrode and lead positions on (A) chest X-ray prior to WiSE-CRT implant and (B) intra-procedure fluoroscopy after WiSE-CRT implant. Surface ECGs during right ventricular pacing (C) and biventricular pacing with the endocardial electrode in the left ventricular septum (D).
7
EHJ-CR-D-21-00403

## Slide 8
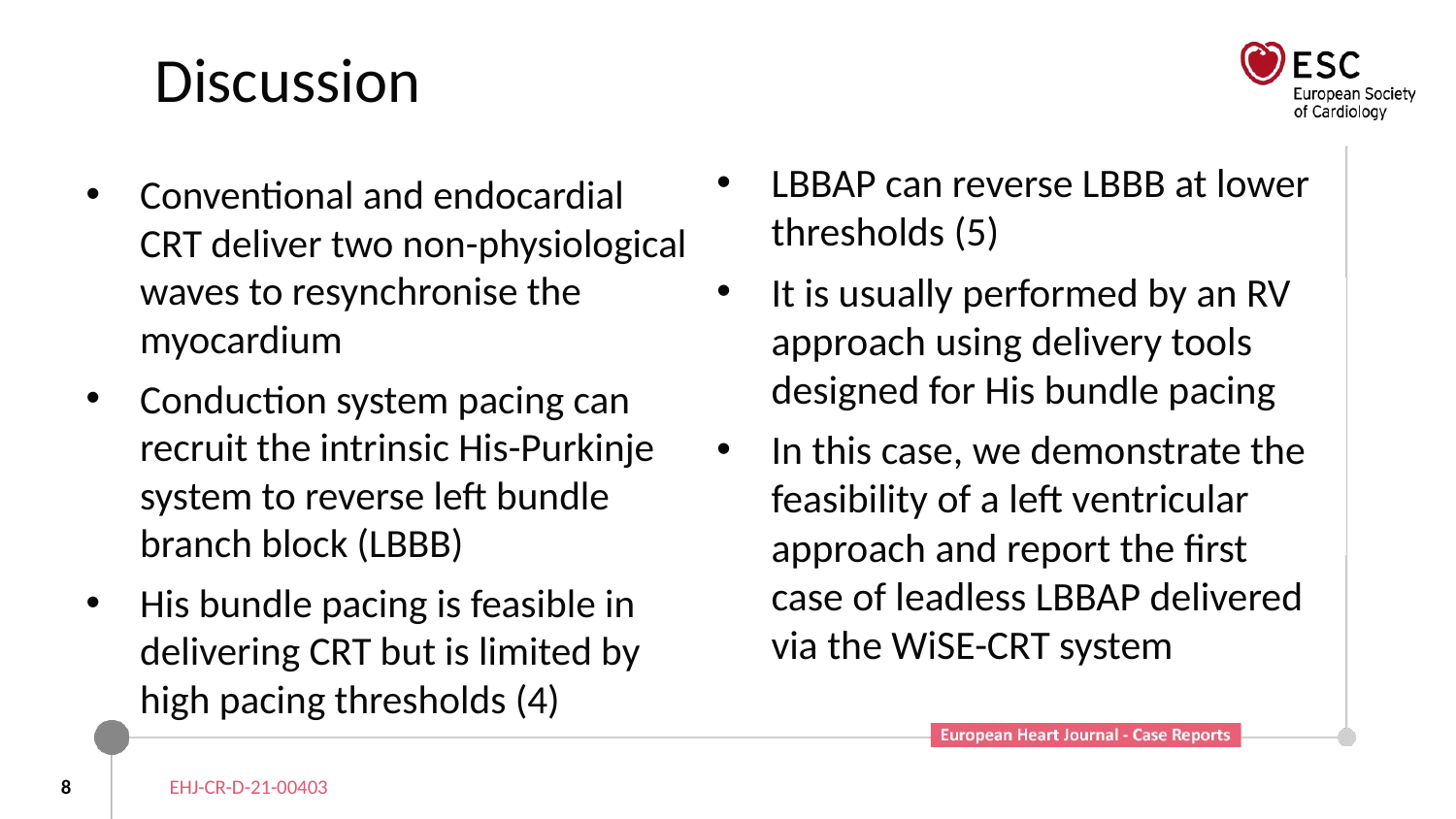

# Discussion
LBBAP can reverse LBBB at lower thresholds (5)
It is usually performed by an RV approach using delivery tools designed for His bundle pacing
In this case, we demonstrate the feasibility of a left ventricular approach and report the first case of leadless LBBAP delivered via the WiSE-CRT system
Conventional and endocardial CRT deliver two non-physiological waves to resynchronise the myocardium
Conduction system pacing can recruit the intrinsic His-Purkinje system to reverse left bundle branch block (LBBB)
His bundle pacing is feasible in delivering CRT but is limited by high pacing thresholds (4)
8
EHJ-CR-D-21-00403

## Slide 9
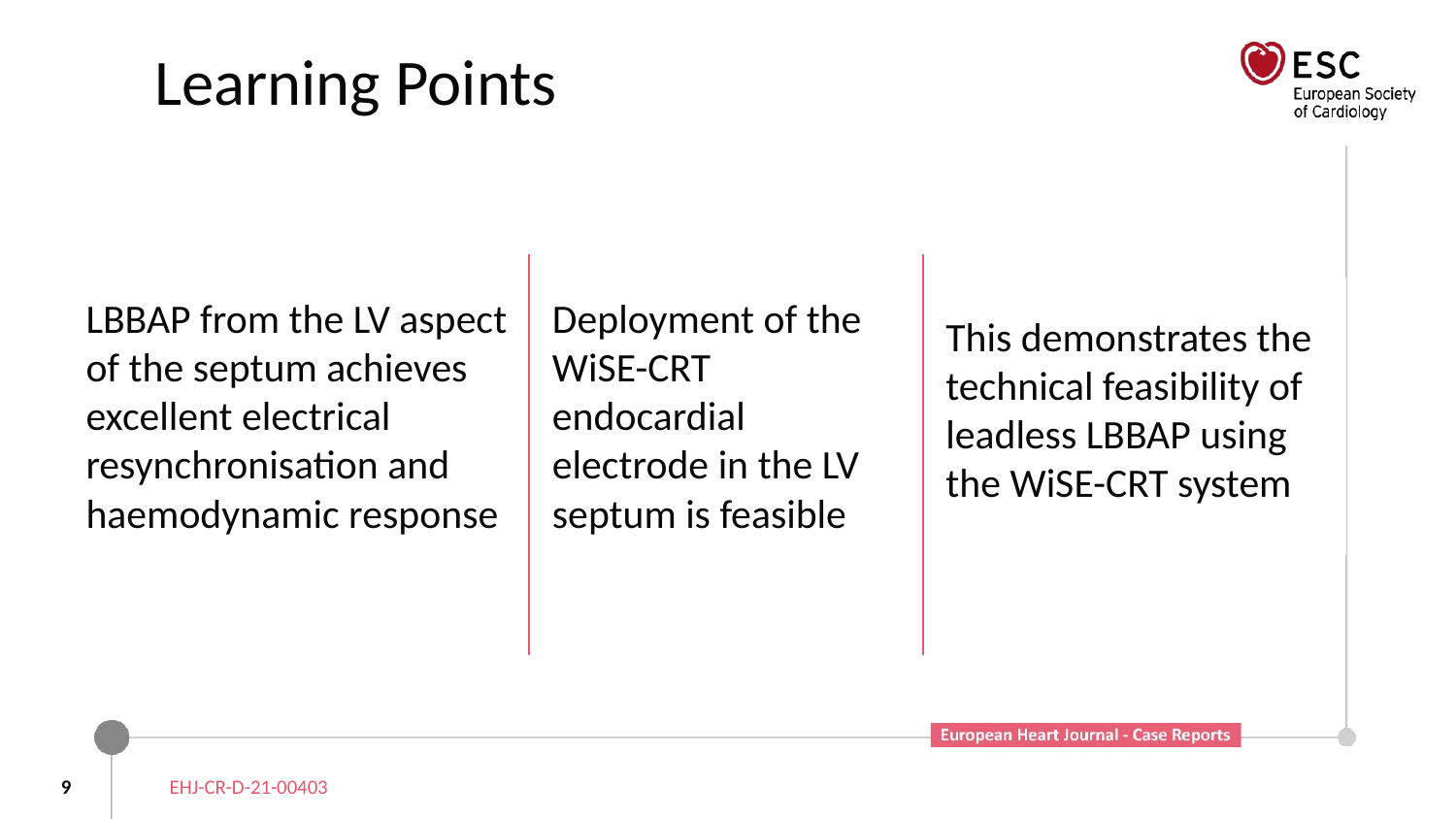

# Learning Points
LBBAP from the LV aspect of the septum achieves excellent electrical resynchronisation and haemodynamic response
Deployment of the WiSE-CRT endocardial electrode in the LV septum is feasible
This demonstrates the technical feasibility of leadless LBBAP using the WiSE-CRT system
9
EHJ-CR-D-21-00403

## Slide 10
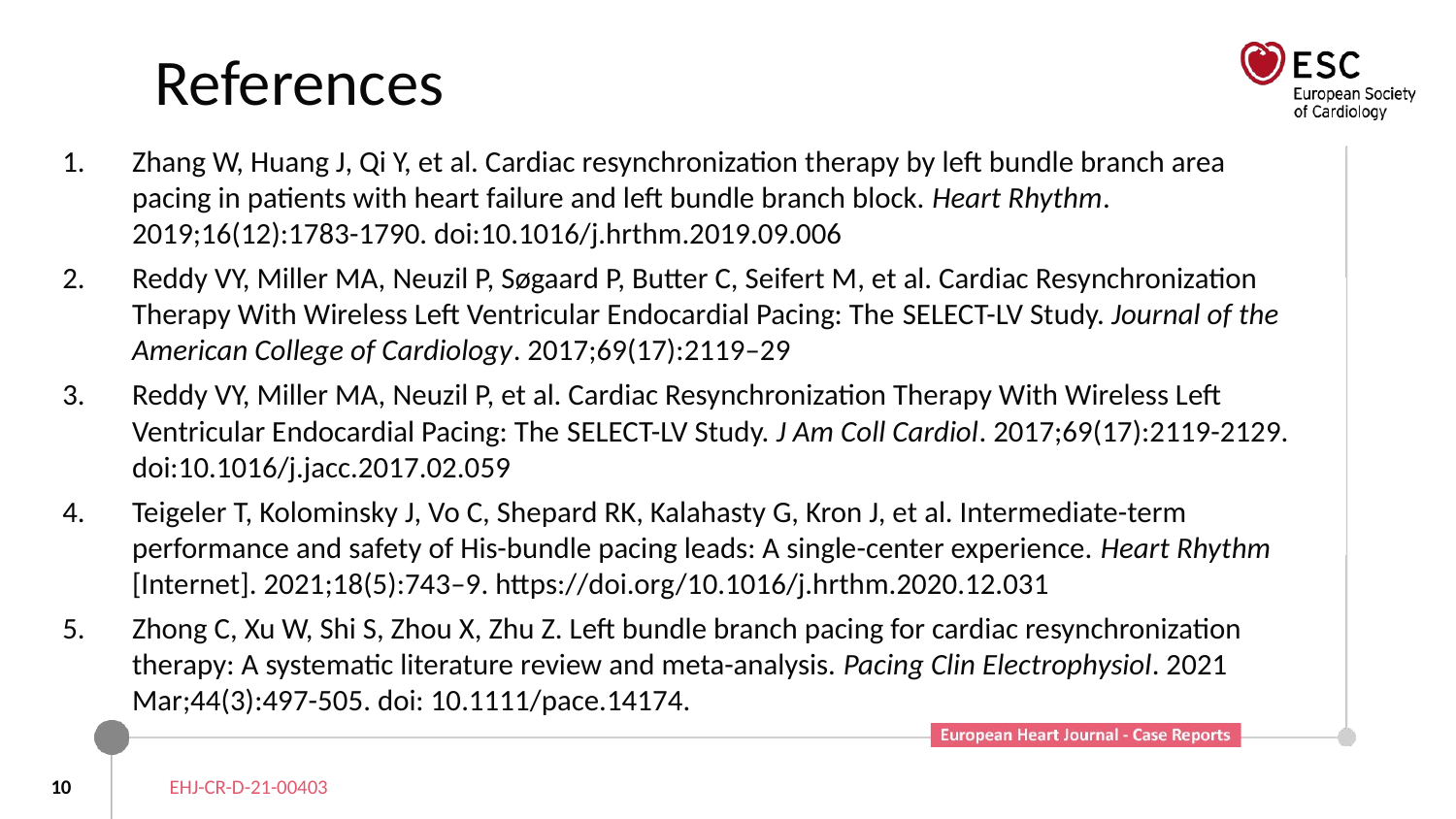

# References
Zhang W, Huang J, Qi Y, et al. Cardiac resynchronization therapy by left bundle branch area pacing in patients with heart failure and left bundle branch block. Heart Rhythm. 2019;16(12):1783-1790. doi:10.1016/j.hrthm.2019.09.006
Reddy VY, Miller MA, Neuzil P, Søgaard P, Butter C, Seifert M, et al. Cardiac Resynchronization Therapy With Wireless Left Ventricular Endocardial Pacing: The SELECT-LV Study. Journal of the American College of Cardiology. 2017;69(17):2119–29
Reddy VY, Miller MA, Neuzil P, et al. Cardiac Resynchronization Therapy With Wireless Left Ventricular Endocardial Pacing: The SELECT-LV Study. J Am Coll Cardiol. 2017;69(17):2119-2129. doi:10.1016/j.jacc.2017.02.059
Teigeler T, Kolominsky J, Vo C, Shepard RK, Kalahasty G, Kron J, et al. Intermediate-term performance and safety of His-bundle pacing leads: A single-center experience. Heart Rhythm [Internet]. 2021;18(5):743–9. https://doi.org/10.1016/j.hrthm.2020.12.031
Zhong C, Xu W, Shi S, Zhou X, Zhu Z. Left bundle branch pacing for cardiac resynchronization therapy: A systematic literature review and meta-analysis. Pacing Clin Electrophysiol. 2021 Mar;44(3):497-505. doi: 10.1111/pace.14174.
10
EHJ-CR-D-21-00403
